# Supplementary figures and images for: Neural mechanisms of emotion during shifting perspectives and recognizing new information: An fMRI study
Source: PLoS One. 2025 Jun 26;20(6):e0309273. doi: 10.1371/journal.pone.0309273 (PMC12200689; doi:10.1371/journal.pone.0309273)

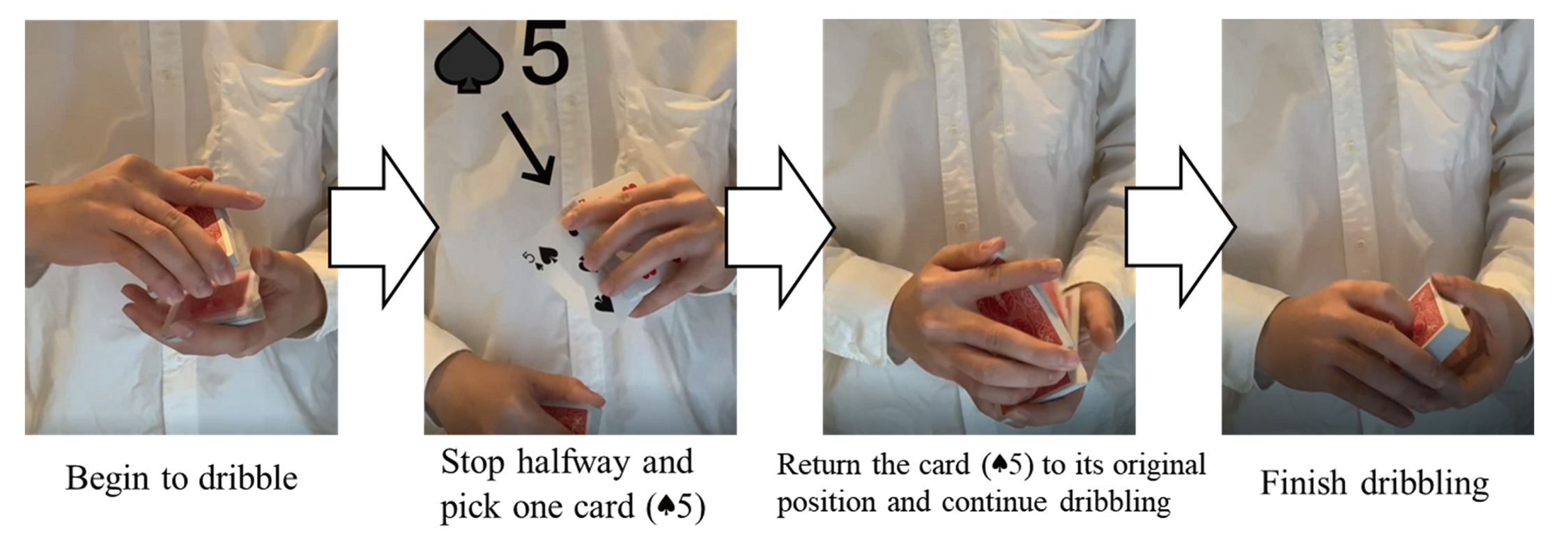

Supplement: S1 Fig — First, the magician held a deck of cards facing down in his right hand. Subsequently, the cards were individually dropped onto the left hand. This technique is known as dribbling (leftmost panel). The dribble stopped midway, and a card was selected and shown to the camera. The card indicated by an arrow was the target card (five of spades) (second panel from the left). The target card was then returned to its original position, and the dribbling continued (third panel from the left). The magic trick ended when the top card dropped onto the left hand (rightmost panel). (TIF) [file pone.0309273.s001.tif]

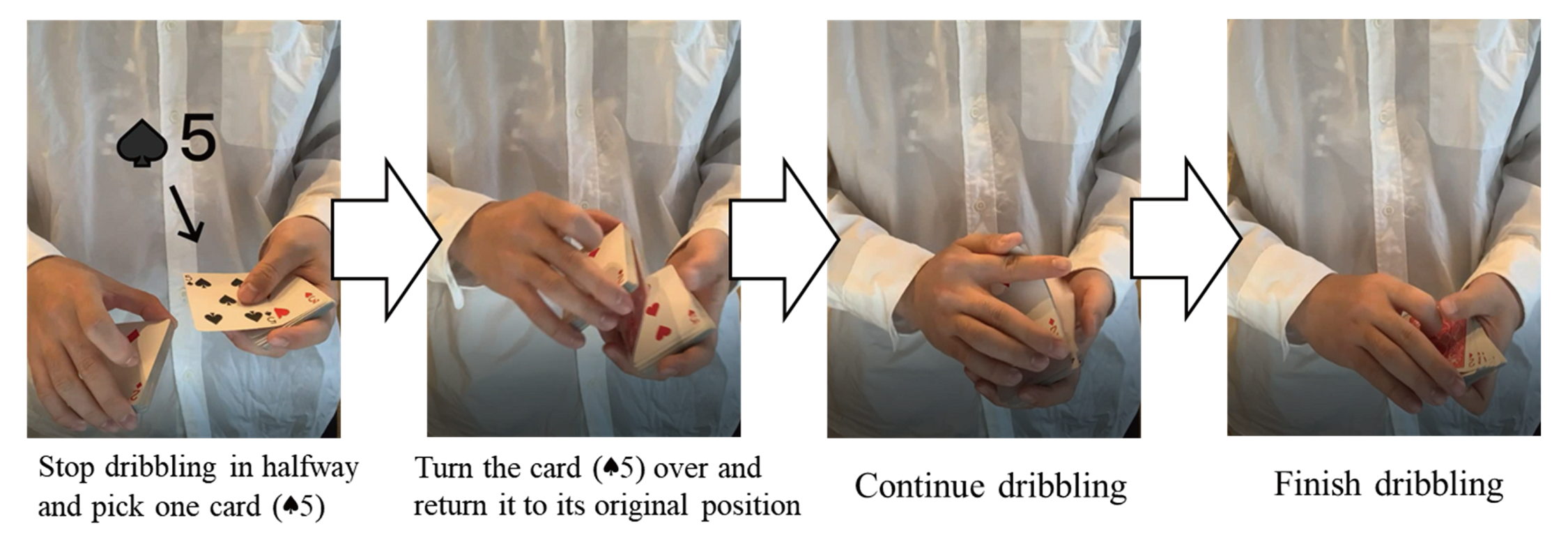

Supplement: S2 Fig — In the high-difficulty condition, participants rewatched Supplementary Video 1. However, in the low-difficulty condition, participants watched the same technique as in Supplementary Video 1, but from a different audience perspective. Supplementary Video 2 for the low-difficulty condition was created so that the final position of the target card could be easily confirmed. Specifically, the target card was selected from the middle of a deck of face-up cards and displayed to the camera (leftmost panel). Next, the target card was turned over and returned to its original position. Only the target card was turned face down (second panel from the left). Subsequently, as shown in Supplementary Video 1, the cards held in the right hand were dropped individually into the left hand using a dribble (third panel from the left). At the end of the video, a face-down card was placed on top of the deck (rightmost panel). This was the target card, which was supposed to be in the middle of the deck; however, it was moved to the topmost position of the deck. (TIF) [file pone.0309273.s002.tif]

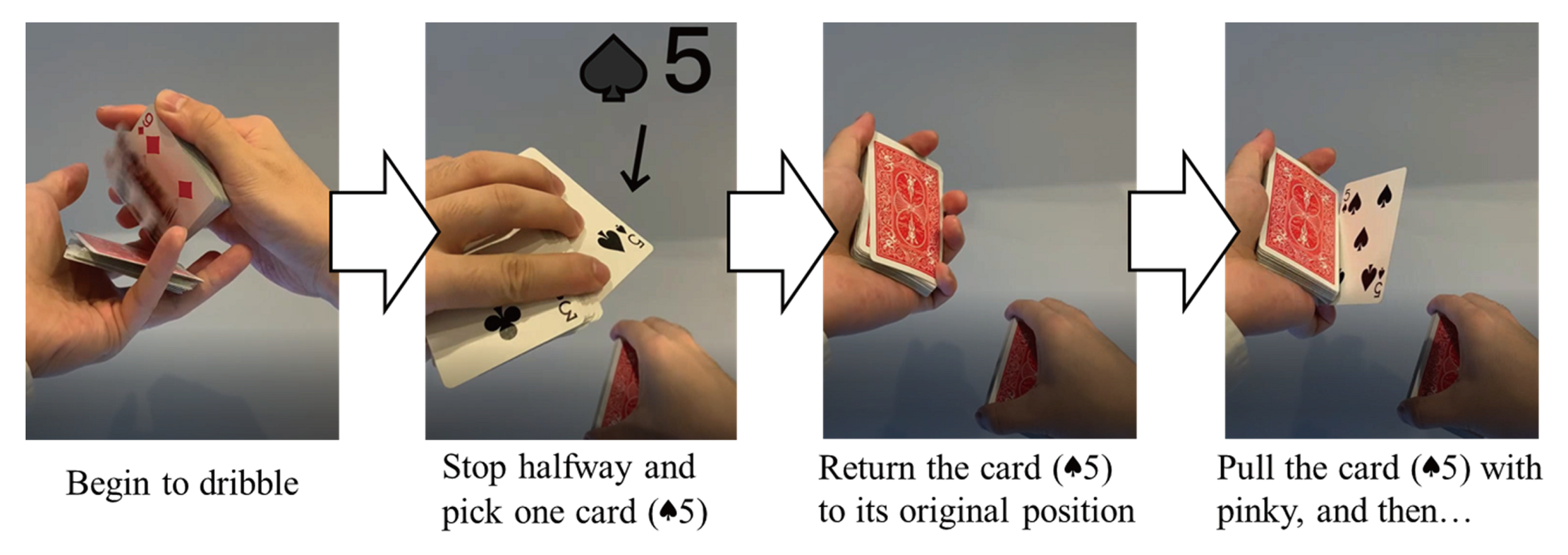

Supplement: S3 Fig — In Supplementary Video 3, a card held in the right hand was dropped individually into the left hand using a card dribble (leftmost panel). Next, the magician stopped midway and selected a card (the target card) to be shown to the camera (second panel from the left). The player then returned the target card to its original position and continued the dribbling (third panel from the left). The technique was the same as that in Supplementary Video 1, except from a different perspective. Just before continuing to dribble, the little finger pulled the target card to a vertical position. Supplementary Video 3 ended at this stage (rightmost panel). (TIF) [file pone.0309273.s003.tif]
