# Supplementary material for: Neural mechanisms of emotion during shifting perspectives and recognizing new information: An fMRI study
Source: PLoS One. 2025 Jun 26;20(6):e0309273. doi: 10.1371/journal.pone.0309273 (PMC12200689; doi:10.1371/journal.pone.0309273)
Supplement: Attachment — Submitted filename: reviewer response.docx [file pone.0309273.s009.docx]

Dear Authors,

Thank you for your thoughtful responses to my comments on the manuscript, PONE-D-24-33040R1: "Neural mechanisms of emotion during shifting perspectives and recognizing new information: An fMRI study.” The revisions you have made effectively address the concerns I raised, and they have significantly strengthened the manuscript. With these improvements, I am pleased to recommend the manuscript for publication.

Congratulations on an exceptional piece of scholarship.

Sincerely
